# Health Professionals' Attitude Toward the Use of Social Media for COVID-19 Related Information in Northwest Ethiopia: A Cross-Sectional Study

**DOI:** 10.3389/fpubh.2022.900293

**Published:** 2022-06-17

**Authors:** Masresha Derese Tegegne, Berhanu Fikadie Endehabtu, Habtamu Alganeh Guadie, Tesfahun Melese Yilma

**Affiliations:** ^1^Department of Health Informatics, Institute of Public Health, College of Medicine and Health Sciences, University of Gondar, Gondar, Ethiopia; ^2^Department of Health Informatics, School of Public Health, College of Medicine and Health Sciences, Bahir Dar University, Bahir Dar, Ethiopia

**Keywords:** social media, attitude, COVID-19, Ethiopia, COVID-19 information

## Abstract

**Background:**

Social media platform is one way to share online information regarding pandemic prevention. However, there is no study regarding the attitude of health professionals toward social media use for the COVID-19-related information. This study aimed to assess health professionals' attitudes toward using social media for COVID-19-related information.

**Methods:**

An institution-based cross-sectional study was conducted among 355 health professionals in Bahir Dar city public health centers, Northwest Ethiopia. A pretested self-administered questionnaire was used to collect the data. The data were entered by EPI-data version 4.6 and analyzed using SPSS version 23 software. Descriptive statistics, bivariable, and multivariable logistic regression analysis were used to describe respondents' attitudes toward using social media for COVID-19 information and identify associated factors. An adjusted odds ratio (*OR*) and a *p*-value with a 95% *CI* were calculated to measure the strength of the association and assess statistical significance.

**Result:**

Out of 341 participants, about 73% of the participants had a good attitude toward the use of social media for COVID-19 information. Age < 24 [adjusted odds ratio (*AOR*) = 3.74, 95% *CI*: (1.53–9.13)] and age group 25–34 years [*AOR* = 2.25, 95% *CI*: (1.04–4.86)], computer training [*AOR* = 2.03, 95% *CI*: (1.03–4.00)], usefulness of social media [*AOR* = 3.25, 95% *CI*: (1.58–6.67)], and trustworthiness [*AOR* = 3.57, 95% *CI*: (1.93–6.60)] were enabling factors for attitude toward the use of social media for COVID-19 related information.

**Conclusion:**

Health professionals had a moderate attitude toward using social media for accessing COVID-19-related information. This implies that after considering positive attitude predictors, such as providing basic computer training, emphasizing the usefulness of social media, and building trusted social media pages, social media platforms can be used as a source of COVID-19-related information for health professionals.

## Introduction

The World Health Organization (WHO) declared coronavirus diseases 2019 (COVID−19) as a public health emergency of international concern on 11 March 2020 (1, 2). Reports reveal that the number of confirmed COVID-19 cases is exponentially increasing (3, 4). As a result of the exponential increase in cases and deaths, the current COVID-19 pandemic is threatening global health. It is vital to prevent pandemics from further spreading in community and healthcare settings through promotion and education. Social media platforms are a means of pandemic prevention and are used by health professionals for communication purposes (5).

Social media is defined as automated communication through mobile and web-based technologies (6). Evidence showed that social media helps in crisis communication, such as natural disasters and pandemics (5), and it also played a significant role during the COVID-19 pandemic (7, 8).

The use of social media in the era of pandemics warrants greater scrutiny because of its consequences for public understanding of COVID-19 issues. The internet and social media are used as reference guides for accessing COVID-19-related information for health professionals (9). Moreover, health professionals can provide access to valid information about the pandemic to the community using social media sites (10).

Evidence suggests that social media platforms encourage the exchange of current information to improve community and healthcare workers' knowledge, practice, and attitude (11). In a previous Egyptian study, more than half of the respondents said that social media is their primary source of COVID-19-related information (12). Similarly, Karasneh et al. (13) found that social media networking sites and the internet were the primary means for sharing COVID-19-related information. Furthermore, an international study has been undertaken on health professionals' attitudes toward using social media for accessing health information (14–18). Jordanian evidence revealed that nursing students were enthusiastic about using social media to access COVID-19 information (15, 19). Similar studies identified that health professionals' use of social media platforms had been considered an efficient instrument for education, exchange, and communication in the COVID-19-related information (20, 21).

According to the study, issues, such as a lack of training, a negative perception of social media sites' usefulness, and a lack of access to the internet and electronic devices contribute to a negative attitude about using social media to receive health information (14, 17). Besides this, the legality and trustworthiness of information shared on social media are limited, resulting in fake news, professional identity, and privacy violations.

Health practitioners were recognized to be on the front lines of raising community awareness and preventing the spread of the COVID-19 pandemics. It is difficult for healthcare providers to teach patients face to face and offer them accurate information about COVID-19 transmission and preventative strategies because of the nature of COVID-19 transmission. The lack of a well-integrated system to combat COVID-19 makes prevention challenging in developing nations (22). According to research, traditional media were revealed to be sources of COVID-19 information in Ethiopia (23). Conventional methods of communication do not reach a large number of people quickly or obtain an immediate reaction. As a result, social media networking platforms have become health professionals' only method of connection in their communities.

As we have seen in different countries of the world, a few studies have explored health professionals' behavior in using social networking sites for accessing COVID-19-related information (15). Similarly, there is limited evidence on health professionals' attitudes regarding using social media platforms to share COVID-19-related information in Ethiopia. As a result, the current study aimed to determine health professionals' attitudes toward using social media for COVID-19-related information and its associated factors in Bahir Dar town health centers in northwestern Ethiopia. The findings of this study could add to our understanding of health professionals' attitudes toward using social media to learn about COVID-19. Our results can also help health administrators, policymakers, and planners in implementing effective pandemic response systems. These initiatives may assist them in implementing new pandemic prevention mechanisms.

## Methods

### Study Design and Setting

A cross-sectional study was carried out among health professionals from 25 January to 20 February 2021, to assess the attitude of health professionals toward the use of social media for accessing COVID-19 related information in Bahir Dar town health centers, which is the objective of this research. Bahir Dar is the capital of the Amhara Region and is situated in the northwestern part of Ethiopia, which is 490 km far from the capital, Addis Ababa (24). In the city, there are 10 health centers, namely, Abay health center, Bahir Dar health center, Minilik Health Center, Han Health Center, Meshenty Health Center, Shimbit Health Center, Shumabo Health Center, Tis Abay Health Center, Zegie Health Center, and Zenzelma Health Center, which serve approximately 750,991 population.

### Study Subject

The population included in this study consisted of all health professionals who had at least one social media account in Bahir Dar town health centers. This population had experience in social media practices and could give adequate data on the research questions. However, health professionals who were severely sick and on annual leave during the data collection period were excluded.

### Sample Size and Sampling Procedure

The calculated sample size for this study using the single population proportion formula was 423, while the total number of health professionals in Bahir Dar town health centers who had at least one social media account was only 355. Therefore, all health professionals meeting the eligibility criteria were included.

### Study Variables and Outcome Measurement

The study's primary outcome variable was health professionals' attitudes toward using social media for COVID-19-related information. This study's tools were adapted from a review of related studies (14, 17). Some independent variables include socio-demographic parameters, technological-related variables, and behavioral variables.

*Attitudes toward social media usage for COVID-19-related information* were measured by using a 5-point Likert scale from “strongly disagree” (score 1) to “strongly agree” (score 5) (14). The item scores for each composite variable were added and divided by the number of items to create a composite variable ranging from scores 1 to 5 for data analysis (25, 26). As a result, final scores of three or more (strongly disagree, disagree, and neutral) were labeled as “Good attitude.” In contrast, final scores of three or less (strongly disagree, disagree, and neutral) were categorized as “Poor attitude” (27).

### Data Collection Tools and Quality Assurance

A self-administered, structured, and pre-tested questionnaire in English was used to collect data from the study population. The data were collected by ten health informatics technicians with excellent communication skills, with two health informatics professionals with prior study experience in supervising the data collection process. In addition, 1 day of training was given to data collectors. A pre-test was done out of the study area, at Gondar town in Gondar health center with 10% of the study population to avoid unclarity before actual data collection. The researchers attempted to reduce response bias based on survey replies during the pre-test. This was done by looking over the questions and making sure they were balanced, avoiding any that would provoke a strong emotional response in one direction or the other. The pre-test results were also used to evaluate the data collection instrument's validity and reliability. Cronbach's alpha result was used to assess the data collection instrument's internal consistency, and the score on attitude toward social media usage for COVID-19 was 0.86.

### Data Processing and Analysis

The data entry and cleaning were performed by using Epi-data version 4.6. After that, the data were exported to SPSS version 25 software for analysis. Descriptive statistics and percentages were computed to describe the socio-demographic variables and attitudes toward social media usage for COVID-19 information. Bivariable and multivariable binary logistic regression analyses were used to measure the association between the dependent and independent variables. In the bivariable regression analysis, factors having a *p*-value of <0.2 were included in the multivariable regression analysis. The odds ratio (*OR*) with 95% *CI* and *p*-value were calculated to evaluate statistical significance. For all statistical significance tests, the cut-off value was *p* <0.05. Multicollinearity assumptions were evaluated before running the logistic regression model, and all the variance inflation factor (VIF) values were <3, indicating no multicollinearity between variables. Since our outcome variable is not normally distributed, we have fitted a multivariable binary logistic regression. The model fitness was assessed by Hosmer and Lemeshow test, which indicates that it was well-fitted where the *p*-value was >5% (i.e., *p*-value = 0.596).

## Results

### Socio-Demographic Characteristics

The questionnaire was completed by 341 (96%) of the 355 participants. The respondents' average age was 31.49 (SD ± 6.42) years. As shown in (Table 1), 235 (68.9%) of health professionals belonged to 25–34 years age group. More than half (54.3%) of the respondents were men. The majority of the responders (61.3%) had a bachelor's degree, and more than half (55.7%) were nurses or midwives. The majority of the respondents, 222 (65.1%), had <5 years of experience with social media networks (SMNs).

**Table 1:** Socio-demographic characteristics and computer and internet access at Bahir Dar city health centers (*n* = 341).

**Socio-demographic characteristics**	**Frequency**	**Percentage**
**Gender**		
Male Female	185 156	54.3 45.7
**Age**		
<24 25–34 >35	36 235 70	10.6 68.9 20.5
**Profession**		
Nurse Midwives Health officer Laboratory Pharmacy Other^*^	123 67 52 46 38 15	36.1 19.6 15.2 13.5 11.1 4.4
**Educational level**		
Diploma BSc. Degree Masters	108 209 24	31.7 61.3 7.0
**Years of experience on social media networks**		
<5 years 6–10 years >10 years	222 73 46	65.1 21.4 13.5

* *Environmental health and nutrition*.

### Computer and Internet Access

Table 2 shows that 321 (94.1%) health professionals owned at least one electronic device, with smartphones 87.1%, laptops 22.9%, and desktops 15%. Of all respondents, 206 (60.4%) have access to the internet, and approximately 48.7% of respondents have basic computer training.

**Table 2:** Computer and internet access among health professionals working at Bahir Dar city health centers (n = 341).

**Computer and internet access**	**Frequency**	**percentage**
Computer access^*^		
Smartphone Laptop Desktop Don't have access	297 78 51 20	87.1 22.9 15.0 5.9
**Internet access**		
Yes No	206 135	60.4 39.6
**A stable power supply to use the internet**		
Yes No	198 143	58.1 41.9
**Did you take any kind of computer training?**		
Yes No	166 175	48.7 51.3

* *Multiple responses possible*.

### The Attitude Toward the Use of Social Media for COVID-19 Related Information by Health Professionals

Of the total respondents, 248 (72.7%) (95% *CI*: 67–77%) had a good attitude toward using social media for accessing COVID-19-related information. Table 3 shows that 43.1% of health professionals believe that using social media to share COVID-19-related information and knowledge is an essential use of time. About 45.7 and 41.9% of the total respondents believe that sharing COVID-19 information and knowledge *via* social media is valuable and engaging. Furthermore, 47.2% of them thought using social media to stay up to date on COVID-19 was a good idea.

**Table 3:** Health professionals' attitude toward the use of social media for COVID-19-related information (*n* = 341).

**Attitude statements**	**SD (%)**	***D* (%)**	***N* (%)**	***A* (%)**	**SA (%)**
Sharing COVID-19 information and knowledge by using social media is an essential use of time	31 (9.1)	54 (15.8)	65 (19.1)	147 (43.1)	44 (12.9)
Sharing COVID-19 information and knowledge by using social media is very beneficial	20 (5.9)	51 (15.0)	55 (16.1)	156 (45.7)	59 (17.3)
Using social media for accessing COVID-19 updates make me very engaging	21 (6.2)	56 (16.4)	86 (25.2)	143 (41.9)	35 (10.3)
Using social media is a good way to get current information for COVID-19	13 (3.8)	32 (9.4)	34 (10.0)	161 (47.2)	101 (29.6)
Using social media is returns high quality information regarding COVID-19	17 (5.0)	64 (18.8)	44 (12.9)	137 (40.2)	79 (23.2)

*SD, strongly disagree; D, disagree; N, neutral; A, agree; SA, strongly agree*.

Of all respondents, 239 (70.1%) healthcare providers were daily users of social media. As shown in Table 4, Facebook (87.1%) and Telegram (59.2%) were found to be the two most widely used platforms for accessing COVID-19-related information among health professionals working at Bahir Dar town health centers. Health professionals' main reasons for using social media to seek COVID-19-related information were to know COVID-19 treatment 174 (51%) and for global and local death reports 177 (51.9%) (Table 4).

**Table 4:** Social media usage for COVID-19-related information in Bahir Dar city health centers (n = 341).

**Frequency of social media use**	**Frequency**	**Percentage**
**Frequency of using social media networks**.		
Daily Not daily	239 102	70.1 29.9
Used platform^*^		
Facebook Telegram WhatsApp YouTube Twitter Others^**^	297 202 70 124 8 19	87.1 59.2 20.5 36.4 2.3 5.5
Reasons for using social media to seek COVID-19^*^		
For diagnosis For treatment For global and death reports For global and local case reports To find updates on the prevention methods To find updates on the mode of transmission	166 174 177 169 139 127	48.7 51.0 51.9 49.6 40.8 37.2
**Privacy concern of while using SMN to seek COVID-19 information**		
Concerned Not concerned	194 147	56.9 43.1
**Trustworthiness of COVID-19 information posted on SMN**		
Trustworthy Not trustworthy	254 87	74.5 25.5
**Usefulness of SMN to seek COVID-19 information**		
Useful Not useful	279 62	81.8 18.2

* *Multiple responses possible*,

** *(Instagram and Tik Tok)*.

While using social media to access COVID-19-related information, about 74.5% of health professionals concluded that COVID-19-related information released on SMNs was trustworthy. Furthermore, 81.8% of health professionals argued that COVID-19-related information published on SMNs was useful, and 194 (56.9%) had privacy concerns while using SMNs for COVID-19-related information.

### Factors Associated With Health Professionals' Attitude Toward Using Social Media for COVID-19 Related Information

In both bivariable and multivariable analysis age, basic computer training, the usefulness of SMN to seek COVID-19-related information, and the trustworthiness of COVID-19 information posted on SMN were significant variables in the attitude of health professionals toward the use of social media for COVID-19 related information (Table 5).

**Table 5:** Factors associated with health professional's attitude toward the use of social media for COVID-19-related information at Bahr Dar city health centers (*n* = 341).

**Characteristics**	**Attitude**		**COR (95% CI)**	**AOR (95% CI)**
	**Good (%)**	**Poor (%)**		
**Age**			
<24 years 25–34 >35 years	85 (24.9) 136 (39.9) 27 (7.9)	21 (6.2) 47 (13.8) 25 (7.3)	3.75 (1.81–7.73) 2.69 (1.41–5.06) 1	3.74 (1.53–9.13)^*^ 2.25 (1.04–4.86)^*^
**Educational level**			
Master's degree BSc Degree Diploma	20 (5.9) 157 (46.0) 71 (20.8)	6 (1.8) 51 (15.0) 36 (10.6)	1.69 (0.62–4.57) 1.56 (0.93–2.60) 1	1.81 (0.48–6.75) 1.20 (0.64–2.25) 1
**Experience on SMN**			
>10 years 6–10 years ≤ 5 years	36 (10.6) 62 (18.2) 150 (44.0)	9 (2.6) 11 (3.2) 73 (21.4)	1.94 (0.89–4.25) 2.74 (1.36–5.52) 1	1.39 (0.56–3.47) 1.95 (0.85–4.46) 1
**Device access**			
Yes No	238 (69.8) 10 (2.9)	83 (24.3) 10 (2.9)	2.86 (1.15–7.13) 1	1.75 (0.55–5.56) 1
**Internet access**			
Yes No	166 (48.7) 82 (24.0)	49 (14.4) 44 (12.9)	1.81 (1.11–2.95) 1	1.01 (0.55–1.88) 1
**Frequency of using SMN**			
Daily Not-daily	189 (55.4) 59 (17.3)	50 (14.7) 43 (12.6)	2.75 (1.66–4.54) 1	1.39 (0.74–2.63)
**Electricity availability**			
Yes No	170 (49.9) 78 (22.9)	47 (13.8) 46 (13.5)	2.13 (1.31–3.47) 1	1.04 (0.56–1.95) 1
**Basic computer training**			
Yes No	143 (41.9) 105 (30.8)	23 (6.7) 70 (20.5)	4.14 (2.43–7.07) 1	2.03 (1.03–4.00)^*^ 1
**Privacy**			
Concerned Not-concerned	137 (40.2) 111 (32.6)	56 (16.4) 37 (10.9)	0.81 (0.50–1.32) 1	0.79 (0.43–1.44) 1
**Trustworthiness**			
Trustworthy Not-trustworthy	210 (61.6) 38 (11.1)	44 (12.9) 49 (14.4)	6.15 (3.60–10.49) 1	3.57 (1.93–6.60)^*^ 1
**Usefulness**			
Useful Not-useful	224 (65.7) 24 (7.0)	55 (16.1) 38 (11.1)	6.44 (3.57–11.63) 1	3.25 (1.58–6.67)^*^ 1

* *Significance at p-value < 0.05*.

Health professionals who were younger (Age <24 and 25–34 years) were approximately [adjusted odds ratio (*AOR*) = 3.74, 95% *CI*: (1.53–9.13)] and [*AOR* = 2.25, 95% *CI*: (1.04–4.86)] times more likely to have a good attitude toward using social media for COVID-19-related information when compared with those who are >35 years of age. Health professionals taking basic computer training were [*AOR* = 2.03, 95% *CI* = (1.03–4.00)] times more likely to have a good attitude toward using social media for COVID-19-related information. Similarly, health professionals who perceived social media as helpful in seeking COVID-19 information were [*AOR* = 3.25, 95% *CI*: (1.58–6.67)] times more likely to have a good attitude toward using social media for COVID-19-related information than those who perceived it not helpful. Respondents argued that COVID-19 information posted on SMN trustworthy were [*AOR* = 3.57, 95% *CI*: (1.93–6.60)] times more likely to show a good attitude toward using social media for COVID-19 related information than their counterparts.

In other words, educational level, experience with SMN, device access, internet access, frequency of using SMN, electricity availability, and privacy concern were found to be non-significant variables (*p*-value > 0.05) in the attitude of health professionals toward using social media for accessing COVID-19-related information (Table 5).

## Discussion

The study aimed to assess health professionals' attitudes toward social media use for COVID-19-related information and its associated factors in Bahir Dar city health centers in Northwestern Ethiopia. As far as we know, this is the first study in Ethiopia to look into health professionals' perspectives on using social media for COVID-19 information.

This study showed that three-fourths (72.7%) of health professionals had a favorable attitude toward using social media for accessing COVID-19-related information in this study. The possible explanation for the high attitude level in this study may be COVID-19 pandemic makes health professionals have a good attitude toward the technology (28). Furthermore, another reason may be that health professionals have more information needed due to the nature of the COVID-19 pandemic (29, 30).

This study showed that Facebook and Telegram were the most frequently used social media platforms for accessing COVID-19-related information. This finding supports previous research (31, 32) that found Facebook and WhatsApp to be the most popular platforms for accessing COVID-19 data. However, in this study, Telegram is more popular than WhatsApp. The difference may be due to cross-country differences in the preferences of social media platforms. Another reason might be that the availability of government-related sites on Telegram forced health professionals to utilize this platform, such as the Ethiopian Public Health Institute.

The multivariable model revealed that respondents' age, basic computer training, the usefulness of social media for accessing COVID-19 information, and the trustworthiness of COVID-19 information posted on social media were all significant factors associated with health professionals' attitudes toward using social media to access COVID-19 related information.

Among the factors, younger health professionals were more likely to have a good attitude toward using social media for COVID-19 information. This conclusion has been supported by previous research on the subject (17, 33). Younger health professionals are more eager to accept and use social media networking sites for COVID-19-related information than older groups. This suggests that older employees need more assistance in adapting to and using social media as sources of COVID-19-related information.

This study also indicated that taking basic computer training was found to have a significant association with the attitude of health professionals toward the use of electronic health information sources, such as social media. This may be justified that computer-related training was more likely to increase health professionals' familiarity with using digital media, including social media platforms for accessing health information. Study findings from Ethiopia (34, 35), Saudi Arabia (36), and the United kingdom (17) support these findings.

The findings also showed that the perceived usefulness of social media networking sites was positively associated with health professionals' attitudes toward using social media to seek COVID-19-related information. This result is consistent with other studies where health professionals who did not believe in the possible advantages of social media were less inclined to use social media for accessing health information (33, 36, 37). This might be because health professionals who believe digital platforms as vital for sharing information may emphasize and seek information from them. This suggests that, in the delivery of health information by social media, there is a need to guarantee that social media will enhance the intended health outcomes.

In this study, respondents who trusted COVID-19 information on SMN were more likely to have a positive attitude toward using social media to seek COVID-19-related information. This finding is consistent with other studies (38). This implies that in the delivery of pandemic information through social media pages, it is better to use more trusted sites than other non-professional pages (34).

As a result, interventions to improve health professionals' basic computer skills, such as training on how to access and use electronic health information sources and emphasizing individual factors, such as usefulness and trustworthiness, may have a positive impact on health professionals' use of social media in combating the COVID-19 pandemic.

## Conclusion

In general, health professionals had a moderate attitude toward using social media for accessing COVID-19-related information. Being young, having computer training, perceived usefulness, and trustworthiness of SMN contributes to a positive attitude toward using social media for COVID-19 information.

Providing comprehensive digital platform training, improving health professionals' expectations of the usefulness of social media networking sites, and implementing more trusted information sources are necessary measures to increase the health professionals' attitude toward using social media for accessing pandemic-related information.

## Limitation

Since this study was an institution-based cross-sectional survey, only health professionals who came during the data collection period were interviewed, thus excluding those who were absent during data collection time. Additionally, the information collected was self-perceived, which might have reporting bias.

## Declaration

### Ethical Considerations

The ethical clearance and approval letter for this study was obtained from the Institutional Review Board (IRB) of the University of Gondar. Communication with officials was made through a formal letter received from the University of Gondar. A supporting letter was also obtained from the Amhara Regional Health bureau. All additional explanations about the research's objective and method were given with the assurance that any information contributed by study participants would be kept private. Written consent was obtained from each study participant. Participation was voluntary, and participants could withdraw from the study if they were not comfortable with the questionnaire.

## Data Availability Statement

The original contributions presented in the study are included in the article/supplementary material, further inquiries can be directed to the corresponding author.

## Ethics Statement

The studies involving human participants were reviewed and approved by Ethical Considerations. The ethical clearance and approval letter for this study was obtained from the Institutional Review Board (IRB) of the University of Gondar. At all level communication with officials was made through a formal letter obtained from the University of Gondar. A supporting letter was also obtained from the Amhara Regional Health bureau. All additional explanations about the purpose of the research and its procedure were explained with the assurance of confidentiality of any information provided by study subjects. Written consent was obtained from each study participant. Participation was voluntary and participants can withdraw from the study at any time if they were not comfortable with the questionnaire. The patients/participants provided their written informed consent to participate in this study.

## Author Contributions

MT made significant contributions in conception, design, data collection supervision, data analysis, interpretation, and write up of the manuscript. TY, BE, and HG have contributed in extensively revising the manuscript, analysis, and interpretation. All authors have approved the final version of this manuscript.

## Conflict of Interest

The authors declare that the research was conducted in the absence of any commercial or financial relationships that could be construed as a potential conflict of interest.

## Publisher's Note

All claims expressed in this article are solely those of the authors and do not necessarily represent those of their affiliated organizations, or those of the publisher, the editors and the reviewers. Any product that may be evaluated in this article, or claim that may be made by its manufacturer, is not guaranteed or endorsed by the publisher.

## Abbreviations

COVID-19: Coronavirus diseases 2019
AOR: Adjusted odds ratio
CI: Confidence interval
Epi-Data: Epidemiological Data
ICT: Information Communication Technology
SMNs: Social media networks
SPSS: Statistical Package for Social Science
WHO: World Health Organization.
